# Molecular Characterization of the *Neuronatin* Gene in the Porcine Placenta

**DOI:** 10.1371/journal.pone.0043325

**Published:** 2012-08-24

**Authors:** Ting Gu, Xi Su, Quanyong Zhou, Xinyun Li, Mei Yu, Yi Ding, Shuhong Zhao, Changchun Li

**Affiliations:** 1 Key Lab of Agricultural Animal Genetics, Breeding, and Reproduction of Ministry of Education and Key Laboratory of Swine Genetics and Breeding of Ministry of Agriculture, Huazhong Agricultural University, Wuhan, People’s Republic of China; 2 Institute of Animal Husbandry and Veterinary, Jiangxi Academy of Agricultural sciences, Nanchang, People’s Republic of China; University of California, Davis, United States of America

## Abstract

Imprinted genes play important roles in placental and embryonic development. *Neuronatin* (*NNAT*), first identified as an imprinted gene in human and mouse brains, played important roles in neuronal differentiation in the brain and in glucose-mediated insulin secretion in pancreatic β cells. In the pig, *NNAT* was reported to be imprinted in eleven tissues. Our previous microarray hybridization study showed that *NNAT* was differentially expressed in Yorkshire and Meishan pig placentas, but the imprinting status and function of *NNAT* in the placenta have not been investigated. We demonstrated for the first time that *NNAT* was monoallelically expressed in the placenta. Immunochemistry analysis showed that NNAT was located in the uterine luminal and glandular epithelium in placentas. We also confirmed the differential expression of *NNAT* in Meishan and Yorkshire pig placentas by qPCR. Using IPA software and the published literature, we created a model network of the possible relationships between *NNAT* and glucose transporter genes. A dual luciferase reporter assay demonstrated that the crucial promoter region of *NNAT* contained a CANNTG sequence in the +210 to +215 positions, which corresponded to the E-box. Our findings demonstrated important roles of *NNAT* in placenta function.

## Introduction

Imprinted genes are a special category of genes that imprinted one allele in the early embryo development decided by the parental origin. The theory raised by Morre and Haig [1] was widely demonstrated that imprinting evolved in mammals because of the conflicting interests of maternal and paternal genes in transferring of nutrients from the mother to her offspring. For example, maternally imprinted genes *Mest* and *Grb10* play important roles in the placental and fetal development of mammals. *Mest* knockout mice were viable and characterized by growth retardation [2]. Mice with a disrupted maternal copy of *Grb10* produced larger embryos and placentas, while mutant mice were 30% larger than normal mice [3].

Chinese Meishan pigs produce 3 to 4 more piglets than Yorkshire pigs in each litter. Numerous investigations have focused on the mechanisms behind this difference. Early investigators believed that factors regulating developmental rate and uniformity of the conceptus were the primary determinants of prolificacy [4]. Further study showed that the weight of Yorkshire placentas dramatically increased from day90 of gestation to term, while in Meishan pigs, the weight of the fetus, not the placenta, increased during this period [5]. These studies indicated that Meishan and Yorkshire pig placentas have different nutrient transport capacities.

We detected differentially expressed genes in the placentas of Meishan and Yorkshire pigs on day75 and day90 of gestation by Affymetrix Porcine Expression Microarray. A total of 226 transcripts on day75 and 577 transcripts on day90 were differentially expressed between placentas from the two divergent breeds. The differentially expressed transcripts included genes involved in angiogenesis, placental development, nutrient transportation and imprinted genes, such as *PEG1*, *PEG3*, *PEG10*, *PLAGL1*, *SLC38A4*, and *DIRAS3* [6]. *Neuronatin (NNAT)* was found to be one of the imprinted genes differentially expressed in Meishan and Yorkshire pig placentas. *NNAT*, also known as paternally expressed gene 5 (*PEG5*), was first discovered in the rat neonatal brain and has significant roles in the differentiation of neurons [7]. Other studies in adipose tissue and pancreatic β cells showed that *NNAT* is involved in adipocyte differentiation and in regulating glucose-mediated insulin secretion [8], [9].

*NNAT* was paternally expressed in human and mouse brains [10], [11]. In the pig, a study showed that *NNAT* was imprinted in 11 tissues, including heart, liver, spleen, lung, kidney, stomach, small intestine, skeletal muscle, fat, uterus, ovary, and pituitary gland [12], but in pig placenta, the expression status inherited from parents was still uncovered to our knowledge.

In this study, we determined the *NNAT* allelic expression status and confirmed the differential expression of *NNAT* in placentas of Meishan and Yorkshire pigs on day75 and day90 of gestation. The published literature was used to create a network model showing the possible relationships between *NNAT* and glucose transporter genes. The crucial promoter region of *NNAT* in JEG-3 and PK-15 cell lines was identified by series analysis of promoter region sequences. This is the first report of *NNAT* expression and regulation of glucose transportation in porcine placenta.

**Figure 1:**
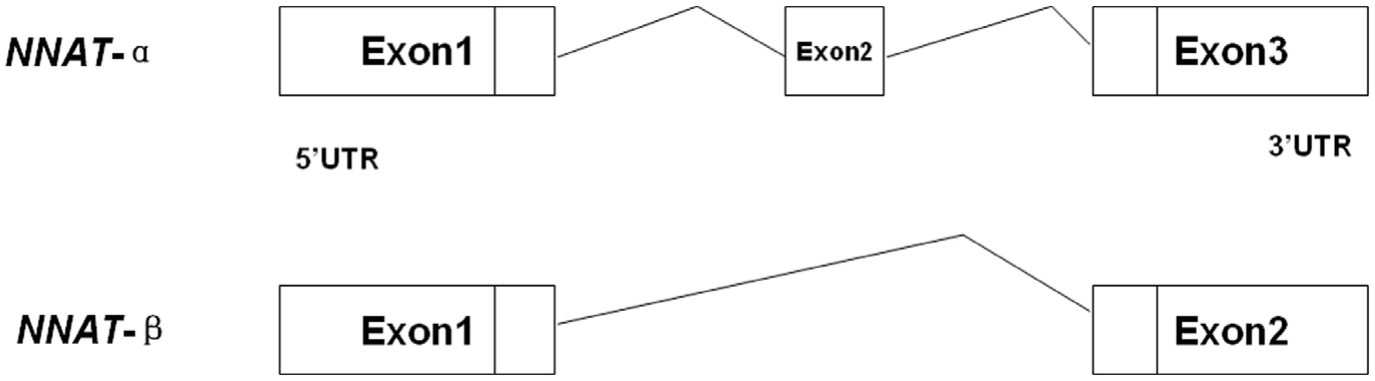
Two transcripts of porcine *NNAT*. *NNAT*-α had three exons, while *NNAT*-β lacked the second exon, which consisted of 81 base pairs.

## Results

### Gene Structure of Porcine *NNAT* and Expression Profiling in Placenta

**Figure 2:**
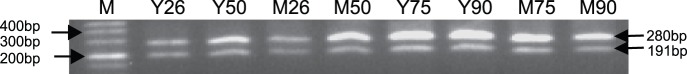
RT-PCR analysis of *NNAT* mRNA in different placentas of different breeds and at different developmental stages. The 280 base pair *NNAT*-α sequence and the 199 base pair *NNAT*-β sequence were both detected in all samples. From left to right, the lane contents were as follows: 500 base pair DNA ladder (Takara), products amplified from the placentas of Yorkshire pigs on day26 and d50 of gestation, Meishan pigs on day26 and day50 of gestation, Yorkshire pigs on day75 and d90 of gestation, and Meishan pigs on day75 and d90 of gestation.

From analysis of the human and mouse *NNAT* sequences, together with the porcine EST analysis, the structure of porcine *NNAT* was determined, as shown in Fig. 1. Two transcripts of NNAT existed in porcine placenta, and both of them were expressed in the placentas of Yorkshire and Meishan pigs at different developmental stages (Fig. 2). The expression level was analyzed by software Quality one. The expression of *NNAT*β and *NNAT*α were decreased in developing placentas in both breeds (Table 1).

**Table 1:** Ratios between *NNAT*α/β.

	Y26	Y50	M26	M50	Y75	Y90	M75	M90
*NNAT*α	7499	11992	6816	15540	21838	21768	16594	13984
*NNAT*β	4595	5526	5938	6874	10012	6746	6183	6246
β/α	0.612	0.46	0.87	0.44	0.45	0.30	0.37	0.44

### Monoallelic Expression of the *NNAT* Gene in the Porcine Placenta

**Figure 3:**
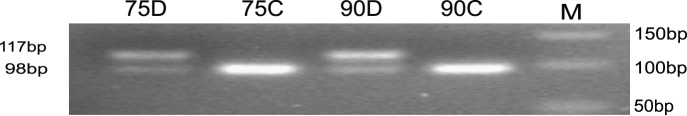
Biallelic status of the *NNAT* gene. PCR products from the genomic DNA (D) and cDNA (C) were digested with *Hin*f- I. Lanes from left to right are as follows: DNA on day75 of gestation, cDNA on day75 of gestation, DNA on day90 of gestation, cDNA on day90 of gestation, and the DL2000 marker.

A SNP in 3′UTR, c.*711C>T was used to detect the biallelic expression status of *NNAT*. When a C was present at the c.*711 position, *Hin*f- I can cut the 117 bp product into two fragments (98 and 19 bp, C-allele), while when a T was present, *Hin*f-I did not cut the PCR product (117 bp, T-allele) (Fig. 3). The genotypes of two pigs (on day75 and d90 of gestation) were CT, while the cDNA expressed only the C allele. This result showed that *NNAT* was also monoallelically expressed in the placentas, besides other reported tissues.

**Figure 4:**
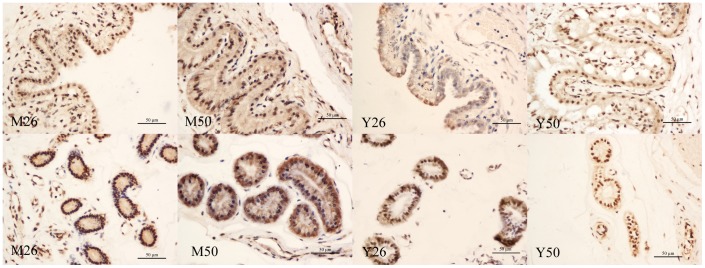
IHC analysis of porcine NNAT in the placenta using rabbit anti-NNAT. Stroma (S) represented the maternal side and fetal chorion (C) represented the fetal side. L: Luminal epithelium; G: glandular epithelium. All images were obtained at 40× magnification. Scale bars represented 50 µm.

### High Expression of NNAT in the Luminal Epithelium and Epithelial Glands of Early Yorkshire and Meishan Pig Placentas

To detect the location of NNAT protein in the placenta, we conducted IHC analysis in the placentas of Meishan and Yorkshire pigs. The luminal epithelium, endometrial fold and fetal chorion of Yorkshire pigs were larger than those of Meishan pigs. Strong NNAT protein signal was detected in luminal and glandular epithelia in Meishan and Yorkshire pig placentas on day26 and d50 of gestation (Fig. 4, dark brown color).

**Figure 5:**
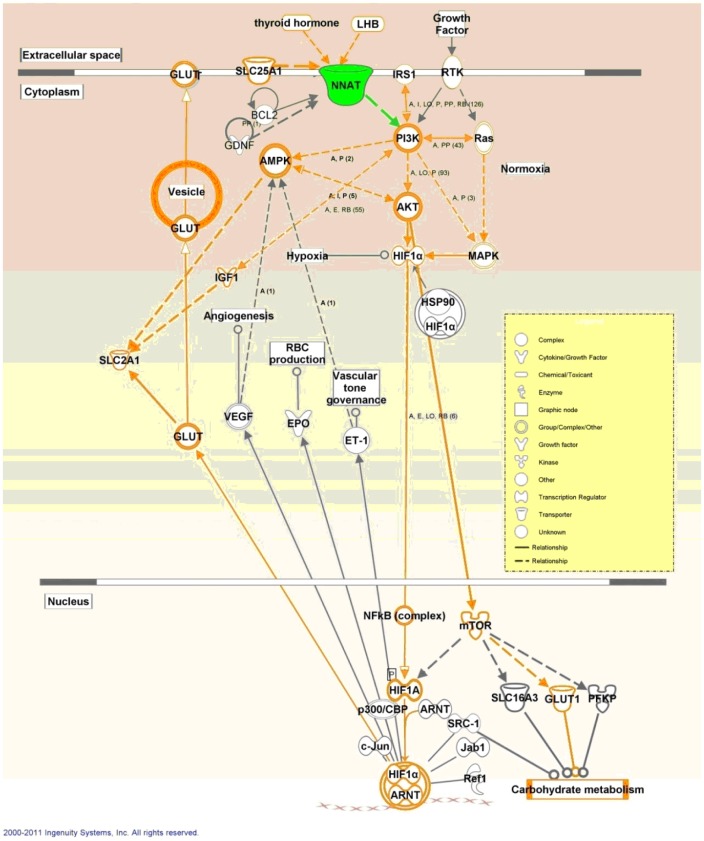
Gene-interaction network analysis of *NNAT*. Solid lines represented direct regulation and dashed lines represent deduced regulation.

**Figure 6:**
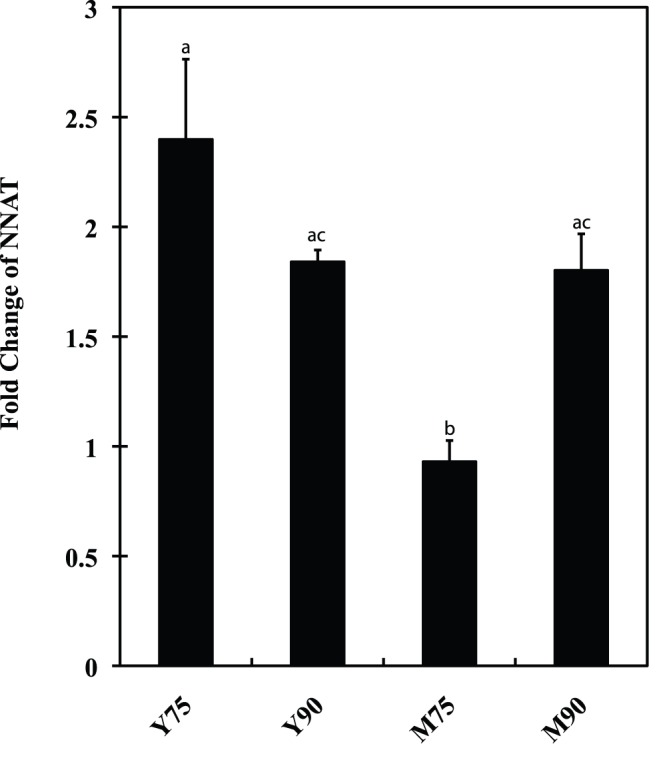
qPCR was used to investigate the mRNA profile of *NNAT* in Yorkshire and Meishan porcine placentas on day75 of gestation (Y75 and M75) and 90 (Y90 and M90). NNAT was significantly lower expressed in M75 compared with M90 and Y75 by SAS Anova test. The different superscript alphanumeric characters indicated a statistically significant difference at p<0.05.

### Gene-interaction Network Construction and qPCR Detection of the mRNA Level in Placentas

**Figure 7:**
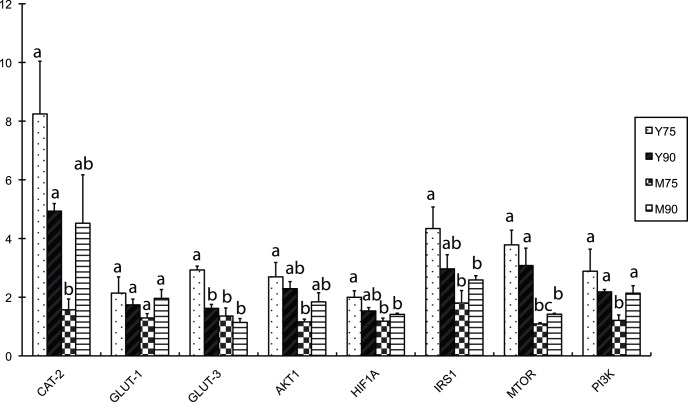
qPCR was used to investigate the mRNA profile of genes in the *PI3K* pathway in Yorkshire and Meishan pig placentas on day75 of gestation (Y75 and M75) and 90 (Y90 and M90). The expression levels were analyzed by SAS Anova test. The different superscript alphanumeric characters indicated a statistically significant difference at p<0.05.

Based on the KEGG prediction and on published papers, a pathway including *GLUT1*, *GLUT3*, *PI3K*, *AKT*, *HIF1A*, *mTOR*, and *IRS* genes that may be affected by *NNAT* in placentas was drawn by Ingenuity Pathway Analysis (IPA) (Fig. 5). qPCR using placental RNAs from the Meishan and Yorkshire pig breeds on day75 and day90 of gestation showed that *NNAT* expression varied with the same pattern as genes in the predicted pathway, indicating that *NNAT* may regulate the transportation of glucose through the *PI3K-AKT* pathway (Figs. 6 and 7).

**Table 2:** Primers for *NNAT* and genes in pathway expression detection.

Primer names	Sequences (5′-3′)	Products size (bp)	Melting temperature
*NNAT*	GGACTAGAGGGCGAGGGTGA	214	62°C
	GGCTTGGAGAAGGTGTTTTGGT		
*CAT2*-Pig	ACAACTGGCGAAGAAGTCCG	100	65°C
	CTGCCGAGACCCCAAAATAG		
*GLUT1*-Pig	GATGAAGGAGGAGTGCCG	106	68°C
	CAGCACCACGGCGATGAGGAT		
*GLUT3*-Pig	TCACGGTCGGTTGAAATG	352	65°C
	CCCACAATCGCTGGAGGA		
*AKT1*-Pig	GAAGCTGCTGGGCAAAGG	207	63°C
	CGGTCGTGGGTCTGGAA		
*HIF1A*-Pig	ACAGAATGGAACGGAGCAAA	119	61°C
	CTTCACAATCATAACTGGTCA		
*IRS1*-Pig	CAACCAGAGTGCCAAAGTGAT	83	61°C
	TGGGTGTCGAGGAGAAGGT		
*MTOR*-Pig	CTGCTCATCAAACAAGCCACATC	116	62°C
	CAAACGCCTCCCGAGAACG		
*PI3K*-Pig	CCAAGAGTTCCGTAAACAGTGCTA	196	63°C
	CTGCATGTAATGGACTGCCTCT		
*GAPDH*-Pig	AAAGGCCATCACCATCTTCC	135	
	GCCCCACCCTTCAAGTGAGCC		
*NNAT*pig2	GGCGGACTCCGAGAGACCA	280/199	58°C
	CCGGTTCTCGACACGGTGTA		
*NNAT*-imprint	CAGCCCCTCACTGATCTTGAAT	117	56°C
	AATCTAGCCGGGGAGACA		

### Promoter Analysis

We cloned a 1766 bp fragment upstream of the transcription starting point using the primers *NNAT*-PS1 and *NNAT*-PR (Table 2). To determine the transcriptional control regions in the *NNAT* promoter, a series of reporter vectors consisting of firefly luciferase regulated by a consecutively truncated promoter were constructed and transfected into porcine kidney 15 cells (PK-15) and the human placental choriocarcinoma cell line JEG3.

**Figure 8:**
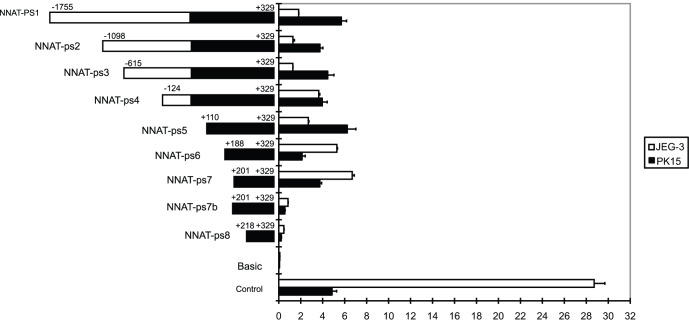
Identification of crucial transcriptional regions of *NNAT*. Three replicates were used for each vector in each transfection. When two base pairs in the region were mutated from CA into AT (NNAT-PS7b) in square box, the transcriptional activity was reduced to a level similar to the basic control.

**Table 3:** Primers used in *NNAT* promoter serious deletion analysis.

Primer name	Primer sequence(5′-3′)	Covering region	Size (bp)
*NNAT*-PS1	CCGCTCGAGCAGCCACTGGAGGCAATA	−1755 to +329	2175
*NNAT*-PS2	CCGCTCGAGCTGCTGGCGGAAGATTG	−1098 to +329	1427
*NNAT*-PS3	CCGCTCGAGGCTCCAAGCACCAGACG	−615 to +329	944
*NNAT*-PS4	CCGCTCGAGCCTGGTTTTTCCATCCGT	−124 to +329	453
*NNAT*-PS5	CCGCTCGAGCGGTATGGAAAGAGCAGA	+110 to +329	219
*NNAT*-PS6	CCGCTCGAGGACCCCCCCAAGTGC	+188 to +329	141
*NNAT*-PS7	CCGCTCGAGGCGCATGCGCAGTTG	+201 to +329	128
*NNAT*-PS7b	CCGCTCGAGGCGCATGCGATGTTG	+201 to +329	128
*NNAT*-PS8	CCGCTCGAGCGGCAGGCGGATACTTA	+218 to +329	111
*NNAT*-PR	CCCAAGCTTTGGTGGTTGAGAGCGAGT		

The protective base pairs were shown in bold. The Xho I site in PS and HindIII site in PR are underlined. Nucleotides in square flames are base pair mutated.

We found that 5′ deletions in a region from +201 to +218 upstream of the promoter (*NNAT*-PS7 to *NNAT*-PS8) almost eliminated its transcription in both the cell lines (Fig. 8). When two base pairs in the +201 to +218 regions were mutated from CA into AT (*NNAT*-PS7b), the transcriptional activity was reduced to a level similar to the basic control. Table 2 showed the mutated base pairs in primers *NNAT*-PS7 and *NNAT*-PS7b in square box.

Dual luciferase promoter activity detection demonstrated that the crucial promoter region of *NNAT* contained an E-box family member binding sequence (CANNTG) located at +210 to +215 was sufficient and necessary for transcription.

## Discussion

*NNAT* was an important gene in regulating mouse brain and adipocyte differentiation and insulin secretion in response to glucose in mouse pancreatic cells [7], [8], [9]. *NNAT* was paternally expressed in human and mouse brains. In pigs, it was reported to be imprinted in eleven tissues. However, there is no study focused on the function of *NNAT* in placentas. We detected *NNAT* expression in placentas. It was of great interest to illustrate possible roles of *NNAT* in the regulation of glucose transportation based on the known roles played by most imprinted genes on fetal and placental development. As indicated by the classic parent-offspring conflict theory, higher expression of paternally expressed *NNAT* would result in a larger fetus in Yorkshire pigs, as the opposite happened in the Mest knockout mice [1]. Our study demonstrated that *NNAT* was monoallelically expressed in porcine placentas and may regulate glucose transportation though the *PI3K-AKT* pathway. The results led us to conclude that *NNAT* was an important imprinted gene in the placenta.

The results of the IHC experiments showed that NNAT was highly expressed in porcine uterine luminal and glandular epithelia. NNAT was expressed in two types of exocrine cells, indicating that this gene may regulate transportation of nutrients. Carbohydrate- and lipid-rich glandular secretions were an important source of nutrients for the fetus when maternal arterial supply to the placenta has not been formed during the early stages of fetal development, and the uterine luminal epithelium mediates transportation between the embryo and mother at this time [13].

The two transcripts of *NNAT* had different functions. *NNAT* was significantly increased in the adipose tissue of ZDF (fat) rats and ectopic expression of *NNAT*α augmented adipocyte by increase adipogenic transcription factors in 3T3-L1 cell line [8]. Study in pancreas showed that *NNAT*α and *NNAT*β increased insulin secretion in the low glucose charge; under chronic high glucose conditions, the ratio of *NNAT*β to *NNAT*α increased in murine pancreatic β-cells, which increased hyperglycemia-induced apoptosis by inhibition of proteasome function. In addition, *NNAT*β decreased the expression of genes important for normal pancreatic β-cell function, such as insulin and glucokinase (*GCK*) [9]. In our study, we found that the expression of *NNAT*α to *NNAT*β had the increased tendency in developing placentas, which suggests that during the development of the placenta, insulin and glucokinase increased, and more glucose was transported into the fetus. Consequently, we assayed the expression of some genes in the *PI3-AKT* pathway and found that the placental expression pattern of these genes during development was similar to that of *NNAT*, indicating *NNAT* may affect or be co-regulated by the insulin and glucokinase pathways in the placenta.

Further, we investigated crucial transcription regions of the *NNAT* gene. Dual luciferase activity detection and basic mutational analysis confirmed that an E-box binding site in the +210 to +215 position of the NNAT promoter region was the crucial transcriptional binding site. This was similar to results in the mouse in which ChIP and EMSA shift experiments in the βTC3 cell line confirmed the existence of an E-box binding site at the −644 to −366 position [14]. These results indicated that the E-box transcription factor was conserved in cell lines from mice, pigs, and humans, but the binding site was species specific.

This is the first report that *NNAT* is expressed in porcine placenta and involved in regulating glucose transportation.

## Materials and Methods

### Tissue Collection

A total of 12 placentas from Meishan and Yorkshire pigs on day75 and d90 of gestation were obtained from a Wen’s Company pig farm. 12 porcine placentas at different stages of gestation (3 each of Meishan and Yorkshire pig placentas on day26 and day50 of gestation) were collected from the swine farm of Huazhong Agricultural University. All sample tissues were either fixed in 4% paraformaldehyde followed by paraffin embedding or stored at −80°C or RNA extraction. All animals involved in this study were conducted according to the regulation (No. 5 proclaim of the Standing Committee of Hubei People’s Congress),which was approved by the Standing Committee of Hubei People’s Congress, and the ethics committee of Huazhong Agricultural University, P. R. China. The approved permit number for this study is “HBAC20091138”.

### Two Different Transcripts of NNAT Expressed on Porcine Placenta

Total RNA was extracted using the RNAprep pure Tissue kit (Tiangen biotech co., Ltd, Beijing, China) and quantified by a Nanodrop ND1000 spectrophotometer. Two micrograms of RNA was reverse-transcribed using M-MLV Reverse Transcriptase (Invitrogen, San Diego, CA) according to the manufacturer’s instructions. RT-PCR was used to detect mRNA of *NNAT* in porcine placentas using the primer pair *NNAT*pig2, as shown in Table 2.

### Biallelic Expression Status Analysis of *NNAT* Genes

cDNA of the porcine *NNAT* gene from three Meishan and three Yorkshire were amplified by PCR and then blasted by NCBI Blast and a SNP in 3′ end was detected. As there is no available commercial enzyme for this SNP, a pair of mutant primers (*NNAT*-imprint in Table 1) that created a *Hin*f- I enzyme site by replacing a C with an A was designed to amplify all individuals’ genomic DNA. The cDNA from the heterozygous samples was amplified and enzyme cut to detect the biallelic expression status. Sequencing was conducted to confirm the genotype.

### Immunohistochemistry (IHC)

IHC to determine NNAT expression in placentas from different pig breeds at 2 gestational time points was performed by standard IHC procedures as described [15]. Three sections of each different sample (5 µm) were deparaffinized and rehydrated in xylene and ethanol. Slides were boiled in citrate buffer (10 mM citrate sodium, 10 mM citric acid, pH = 6.0) in a microwave oven and then cooled to room temperature twice to retrieve antigen. Sections were then incubated with 3% H_2_O_2_ in methanol for 10 min to quench endogenous peroxidase and then in a normal goat serum blocking solution for 20 min. Sections were incubated with the polyclonal primary antibody against NNAT (Abcam Inc., MA, USA) at 4°C overnight and then in biotinylated secondary antibody. Sections were counterstained with hematoxylin and mounted. For each sample, a negative control was performed by replacing the primary antibody with PBS buffer.

### Quantitative RT-PCR (qPCR) Analysis

qPCR was carried out on the LightCycler 480 Real-Time PCR machine (Roche Diagnostics Ltd., Forrentrasse, Switzerland) using Thunderbird SYBR qPCR Mix (Toyobo co., ltd, Osaka, Japan). PCR conditions were as follows: single cycle of 5 min at 95°C, followed by 40 cycles of 30 sec at 95°C, 20 sec at 62°C, and 15 sec at 72°C. The primers were designed by the Primer 6.0 program. The primer sequences, melting temperature and product size were shown in Table 1. The *GAPDH* gene was used as a control. The ANOVA test in the SAS 8.1 program was used to perform statistical analysis.

### pGL3-*NNAT*-PS Series Reporter Construction, Transfection, and Dual-luciferase Reporter Assay

A total of 16 pGL3-*NNAT*luc+vectors were constructed and transfected into PK-15 and JEG-3 cell lines as described previously [16]. A successive series of fragments covering −1766 to+329 of the 5′-flanking promoter of the porcine *NNAT* gene were amplified using the primers in Table 3 and the high fidelity DNA polymerase KOD-Plus (Toyobo) and then inserted into the XhoI/HindIII sites of the pGL3-Basic Vector (Promega, Madison, USA) to construct the pGL3-*NNAT*luc+reporters. These vectors were transfected into PK-15 cells and the human placental choriocarcinoma cell line JEG3 by Lipofectamine 2000 (Invitrogen). The activity of the promoter vectors was detected by a Dual-Luciferase Reporter Assay System (Promega).
